# Identification of CXCL11 as part of chemokine network controlling skeletal muscle development

**DOI:** 10.1007/s00441-020-03398-0

**Published:** 2021-01-27

**Authors:** Malte Puchert, Christian Koch, Konstanze Zieger, Jürgen Engele

**Affiliations:** Institute of Anatomy, Medical Faculty, University of Leipzig, Liebigstr.13, 04103 Leipzig, Germany

**Keywords:** Myoblasts, Skeletal muscle fibres, CXCL11, CXCR3, CXCR7

## Abstract

The chemokine, CXCL12, and its receptors, CXCR4 and CXCR7, play pivotal roles during development and maintenance of limb muscles. CXCR7 additionally binds CXCL11, which uses CXCR3 as its prime receptor. Based on this cross-talk, we investigate whether CXCL11 would likewise affect development and/or function of skeletal muscles. Western blotting and immunolabelling demonstrated the developmentally restricted expression of CXCL11 in rat limb muscles, which was contrasted by the continuous expression of its receptors in proliferating and differentiating C2C12 cells as well as in late embryonic to adult rat limb muscle fibres. Consistent with a prime role in muscle formation, functional studies identified CXCL11 as a potent chemoattractant for undifferentiated C2C12 cells and further showed that CXCL11 does neither affect myoblast proliferation and differentiation nor metabolic/catabolic pathways in formed myotubes. The use of selective receptor antagonists unravelled complementary effects of CXCL11 and CXCL12 on C2C12 cell migration, which either require CXCR3/CXCR7 or CXCR4, respectively. Our findings provide new insights into the chemokine network controlling skeletal muscle development and function and, thus, might provide a base for future therapies of muscular diseases.

## Introduction

Chemokines are chemoattractant cytokines primarily recognized as mediators of various immune responses (Laufer and Legler 2018). Distinct chemokines, such as CXCL12/SDF-1, further control aspects of development, disease progression and regeneration of numerous organs and tissues (Murphy and Heusinkveld 2018). In skeletal muscle, CXCL12 serves as the prime chemoattractant for muscle stem/progenitor cells during myogenesis as well as during regeneration of injured muscles (Puchert and Engele 2014, for review). By affecting anabolic and catabolic pathways, CXCL12 further controls maintenance and growth of adult skeletal muscles/muscle fibres (Puchert et al. 2016), a process eventually allowing to counteract the loss of muscle mass in cancer-induced cachexia (Martinelli et al. 2016). CXCL12 binds to chemokine receptors CXCR4 and CXCR7/ACKR3. However, the presently available data point to CXCR4 as the prime mediator of CXCL12-signalling in skeletal muscles (Hunger et al. 2012; Puchert et al. 2016; Kowalski et al. 2016). CXCR7 additionally binds CXCL11/“interferon-inducible T cell alpha chemoattractant” (I-TAC), which in turn uses CXCR3 as a second receptor (Singh et al. 2013). CXCL11 primarily acts as an inflammatory chemokine, which is induced by interferon-γ at sites of inflammation and attracts CXCR3- and CXCR7-positive immune cells to the site of injury (Singh et al. 2013; Müller et al. 2010). Although of potential therapeutic interest our present knowledge on the function of CXCL11 and its receptors, CXCR3 and CXCR7, in development, maintenance and regeneration of skeletal muscles is limited. Previous work so far demonstrated the increased expression of CXCL11 in muscles of patients suffering from dermatomyositis and Duchenne muscular dystrophy (DMD) (Fall et al. 2005; Raju et al. 2005; De Paepe et al. 2012) as well as the disease-related increase in muscular CXCR3 expression, which depending on the pathological condition either occurs in muscle fibres and/or inflammatory cells within muscles (De Paepe et al. 2005; Feferman et al. 2005; Raju et al. 2003). Indicative for a potential role in myogenesis, CXCL11 expression was further found to transiently increase in mouse myoblasts differentiating in vitro (Griffin et al. 2010).

In the present study, we test whether CXCL11 affects development and/or maintenance of skeletal muscles and if so whether putative effects involve CXCR3 or CXCR7. We analyzed differentiating C2C12 myoblasts/myogenic stem cells as well as developing hind limb muscles for expression of CXCL11 and its receptors, CXCR3 and CXCR7. We further characterized CXCL11 for effects on C2C12 cell proliferation, migration and differentiation as well as on the activity of anabolic and catabolic pathways in the absence and presence of CXCR3 and CXCR7 antagonists. We provide evidence that CXCL11-dependent activation of CXCR3 and CXCR7 is pivotal for the recruitment of myoblasts/muscular stem cells to developing skeletal muscles.

## Materials and methods

### Animals and collection of tissue

Hindlimb muscles (quadriceps muscle) were dissected from Sprague-Dawley rats (Charles River, Sulzfeld, Germany) at embryonic day (E)18, postnatal day 3 (P3) as well as from adult animals (> 4 months). All procedures were carried out according to EU Directive 2010/63/EU. Removal of tissue was approved by local authorities (T16/16). For Western blot analysis, muscle tissue was immediately snap frozen in liquid nitrogen and stored at − 80 °C. For immunohistochemical analysis, excised muscles were stored at 4 °C in PBS containing 20% (w/v) saccharose, transferred to Tissue Tek (Sakura Finetek, Alphen aan den Rijn, Netherlands) and frozen in liquid nitrogen. Material was sectioned on a cryostate (12 µm) and slices were mounted on L-lysine-coated coverslips.

### C2C12 cells

Murine C2C12 myoblasts (CRL-1772, ATCC) were cultured in DMEM (Lonza; Basel, Switzerland) supplemented with 10% fetal calf serum (FCS; Gibco^®^Invitrogen, Carlsbad, CA). For induction of differentiation into myotubes, subconfluent cultures were switched to DMEM containing 1% FCS. C2C12 myotubes were treated with either CXCL11 (100 ng/ml; BioLegend, San Diego, CA) or CXCL12 (100 ng/ml; Almac, East Lothian, Scotland) as specified in the text. At the indicated times, cultures were either processed for Western blot analysis as described below or fixed with 4% paraformaldehyde for subsequent immunolabelling.

### Western blot analysis

Muscle tissue and cultured cells were lysed by ultrasonification in 62.5 mM Tris-HCl (pH 7.3), containing 2% SDS and 10% sucrose. Proteins were denatured at 95 °C for 10 min and further diluted in sample buffer (500 mM Tris–HCl, pH 6.8, containing 4% SDS, 10% glycerol and 2% ß-mercaptoethanol). For detection of phosphorylated proteins, sample buffer was additionally supplemented with sodium orthovanadate (10 mM; Sigma-Aldrich). Proteins (10–20 µg/lane) were separated by SDS-(10%) polyacrylamide gel electrophoresis and transferred to nitrocellulose by electroblotting. Blots were incubated overnight at 4 °C with rabbit or goat anti-CXCL11 (orb13425, Biorbyt, Cambridge, UK; 1:1000; R&D Systems, Cat. No AF572; 1:2000), rabbit anti-CXCR7 (AP17961PU-N, Acris, Herford, Germany; 1:1000), mouse or rabbit anti-CXCR3 (bs-2209R, Bioss, Aachen, Germany; 1:1000; clone No. 49801, R&D Systems, Minneapolis, MN; 1:1000) and goat anti-CXCR4 (ab1670, Abcam; 1:1000); mouse anti-MyHc (MF20, DSHB, Iowa City, IA; 1:50; MF 20 was deposited to the DSHB by Fischman, D.A, DSHB Hybridoma Product MF 20), mouse monoclonal anti-myogenin (SC-12732, Santa Cruz Biotechnology; 1:1000), rabbit anti-MuRF-1 (sc-32920, Santa Cruz Biotechnology; 1:500), or phosphospecific rabbit anti-(p)p70S6K (Thr389: ab78413, Abcam; 1:1000). Antibody labelling was visualized with Super Signal Chemiluminescent Substrates (Thermo Scientific; Waltham, MA). To control for protein loading, membranes were reblotted with mouse anti-GAPDH (10R-G109A, Fitzgerald Industries International, Acton, MA; 1:5000), rabbit anti-ß-actin (13E5; Cell Signaling, Danvers, MA; 1:1000), anti-Erk (No, 4695, Cell Signaling; 1:1000), or rabbit anti-p70S6K (ab32529, Abcam; 1:1000) antibodies. Integrated optical densities of immunoreactive protein bands were measured using Gel-Pro Analyzer software 3.1. (Media Cybernetics, Rockville, MD) and obtained values were corrected for protein loading as appropriate.

### Immunolabelling

For immunolabelling, cultured cells or histological sections were permeablized with saponin (0.05% in PBS) for 10 min and non-specific binding sites were blocked with 5% (v/v) normal donkey serum (GIBCO^®^Invitrogen) in PBS for 1 h. Cultures or slices were incubated with mouse anti-MyHc (MF20, DSHB; 1:25) antibody and either rabbit anti-CXCL11 (Biorbyt, 1:100), goat anti-CXCR4 (ab1670, Abcam; 1:100), rabbit anti-CXCR7 (AP17961PU-N; Acris; 1:100), rabbit anti-CXCR3 (Bioss; bs-2209R; 1:100), or mouse monoclonal anti CXCR3 (clone No. 49801, R&D Systems; 1:100) antibodies overnight in a humid chamber at 4 °C. Primary antibodies were detected by incubating slices for 2 h at room temperature with appropriate Alexa 488-labelled and Alexa 555-labelled secondary antibodies (1:200; Invitrogen, Carlsbad, CA). Cell nuclei were counterstained with DAPI (AAT Bioquest, Sunnyvale, CA) and sections were mounted with Dako glycergel. Sections were examined under a Zeiss confocal laser scan microscope (LSM).

### Chemotaxis

The chemotactic response of C2C12 cells to CXCL11 (100 ng/ml) and CXCL12 (100 ng/ml) was evaluated in a modified 48-well Boyden chamber (Neuro Probe, Cabin John, MD) in which the upper and lower wells were separated by polyornithin-coated Nucleopore^®^ PVP-free polycarbonate filters (Corning, Acton, MA, USA) with 8-µm pore size. For a detailed description of the method see Lipfert et al. (2013). One hour prior to analysis, cultured cells were treated with either the CXCR3 antagonist, AMG487 (10 µM; Tocris, Wiesbaden, Germany; dissolved in DMSO), the CXCR4 antagonist, AMD3100 (10 µM; Sigma, dissolved in double-distilled water), or the CXCR7 antagonist, CCX771 (100 nM; ChemoCentryx; Mountain View, CA; dissolved in DMSO). For control purposes, all cultures received adequate concentrations of DMSO. Numbers of migrated cells were assessed following a 4 h-incubation at 37 °C in a water-saturated atmosphere of 95% air and 5% CO_2_. For quantification, migrated cells were stained with DAPI. Migration index was defined as the ratio of cells migrating in the presence and absence of the chemokine.

### Cell proliferation

To assess mitogenic effects, subconfluent cultures of C2C12 cells were switched to serum-free or low serum (1%) DMEM and immediately treated with CXCL11 (100 ng/ml). After 24 h, cells were incubated with BrDU (10 µM; FITC BrdU Flow Kit; Becton Dickinson Bioscience; cat.-no., 559,619) for 24 h. Cells were subsequently labelled with antibodies against BrdU as well as with 7-aminoactinomycin d (7-AAD) according to the manufacturer’s instructions and subjected to FACS (LSR II Flow Cytometer; Becton Dickinson). Data were analyzed using the FlowJo software version 10 (Treestar). Mitogenic effects were further characterized by assessing mRNA levels of the proliferation marker, Ki67, by quantitative real-time PCR, according to a previously published protocol (Puchert et al. 2020). Gene expression was calculated by the 2(-ΔΔ) CT method and normalized to elongation factor 1$\alpha$ (EF-1$\alpha$). The following primers were used: Mouse Ki67, forward 5′-TGTGAGGCTGAGACATGGAG-3′, reverse. 5′ CCTTGATGGTTCCTTTCCAA-3′.

Mouse EF-1$\alpha$, forward 5′-AGCTTCTCTGACTACCCTCCACTT-3′, reverse 5′-GACCGTTCTTCCACCACTGATT-3′; (both from Eurofins, Ebersberg, Germany).

### RNA interference

Predesigned mouse CXCR3 (ID s64087), CXCL11 (ID s80043) and control (cat.-No. 4390844) siRNAs were purchased from Thermo Fisher (Darmstadt, Germany). Transfection was performed in the presence of serum-free medium using the Lipofectamine RNAiMAX Transfection Reagent (Thermo Fisher) for 16 h. Cells were further cultured in DMEM containing 10% FCS and taken into experiment after 24 or 48 h. Success of RNA interference was validated by Western blotting.

### Statistics

Data, obtained from at least three independent experiments, are given as mean ± SD. Student’s *t* test or one-way analysis of variance (ANOVA) followed by pairwise multiple comparison procedures (Tukey HSD) was used for statistical analysis as appropriate. Differences with *p* < 0.05 were considered significant.

## Results

### Confirming specificity of antibodies in C2C12 cells

C2C12 cells are an established model system for studying mechanisms of myogenesis. They exhibit features of proliferating myogenic progenitors/myoblasts when maintained in high-serum (10%) DMEM (proliferation condition) and undergo myogenic differentiation characterized by myogenin and MyHC expression and the formation of myotubes when maintained with low-serum (1%) DMEM (differentiation condition; see for example Hunger et al. 2012; Supplementary Fig. 4). The CXCR3 antibody used in the present study recognized a major protein band of appropriate size of 40 kD (Supplementary Fig. 1a). Specificity of the CXCR3 antibody was further confirmed in C2C12 cells following transfection with CXCR3 siRNA (Supplementary Fig. 1b). Likewise, specificity of CXCL11 antibodies was confirmed by RNA interference (Supplementary Fig. 1). Due to unknown reasons, the size of the confirmed CXCL11 protein band (approximately 40 kD) is distinctly higher than predicted. Specificity of the CXCR7 and CXCR4 antibodies was successfully established by previous work, using cells with deleted or inhibited chemokine receptor expression (Puchert et al. 2018; Hunger et al. 2012).

**Fig. 1:**
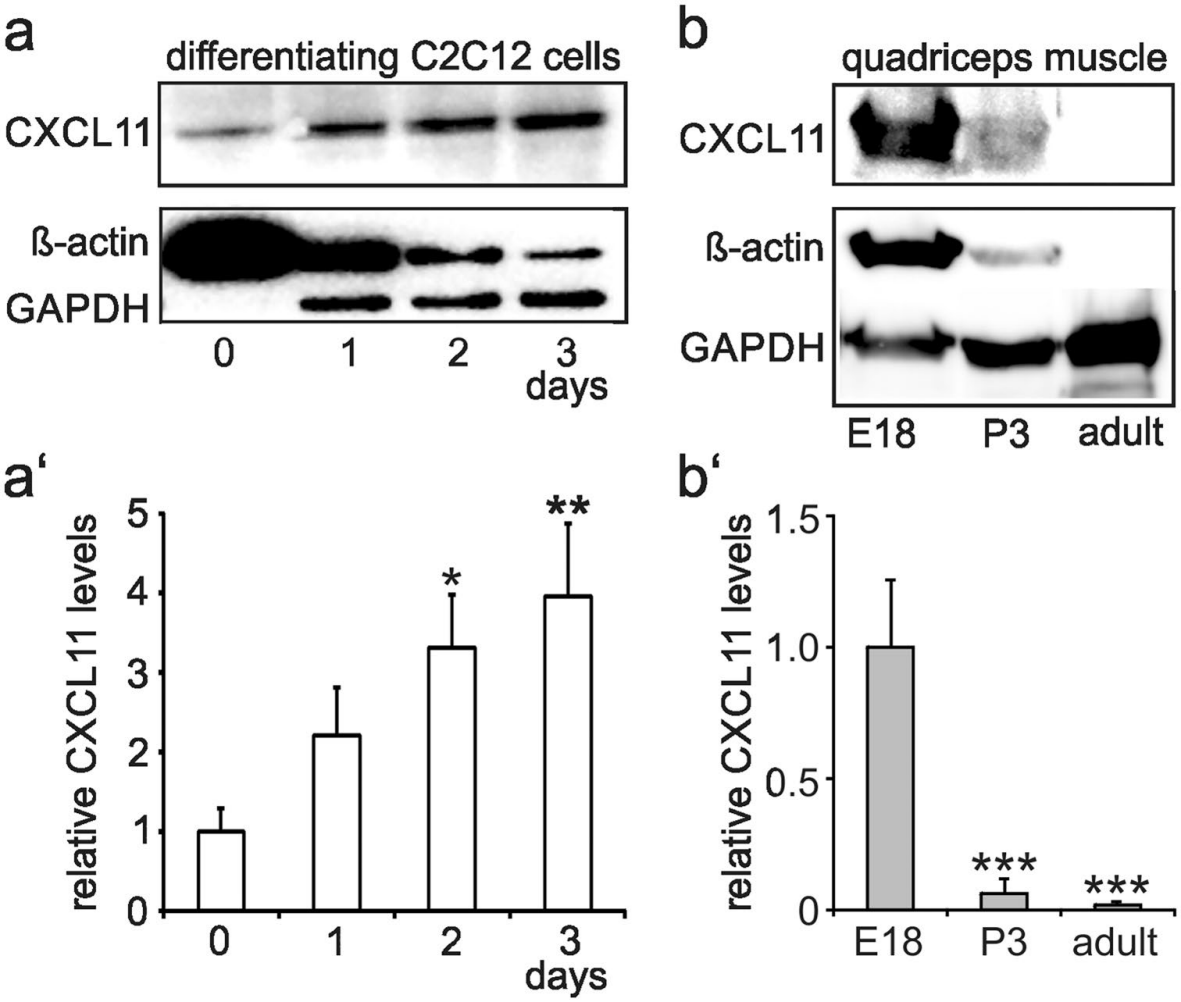
Expression of CXCL11 during myogenesis. **a** Western blot analysis demonstrated that CXCL11 levels gradually increase in C2C12 cells following the switch to differentiation conditions (DMEM + 1% FCS). **a′** Relative levels of CXCL11 (mean ± SD, *n* = 3) as determined by densitometric analysis of immunoreactive protein bands in C2C12 cells maintained under differentiation conditions for the indicated times. CXCL11 levels present prior to the switch to differentiation conditions (day 0) were set to 1. *p < 0.05, **p < 0.005, respective time point vs. day 0. **b** CXCL11 expression is readily detectable by Western blot analysis in the rat quadriceps muscle at E18 with a clear decline into adulthood. **b′** relative levels of CXCL11 (mean ± SD) as determined by densitometry of immunoreactive protein bands from 3 independent experiments. ***p < 0.001, respective time point vs. E18. Note in **a** and **b** that ß-actin as well as GAPDH, which are routinely used as loading controls for Western blotting, are developmentally regulated in muscle cells/fibres. Due to this fact, all samples were carefully adjusted to identical protein levels prior to analysis

### Expression of CXCL11 in developing skeletal muscles

**Fig. 2:**
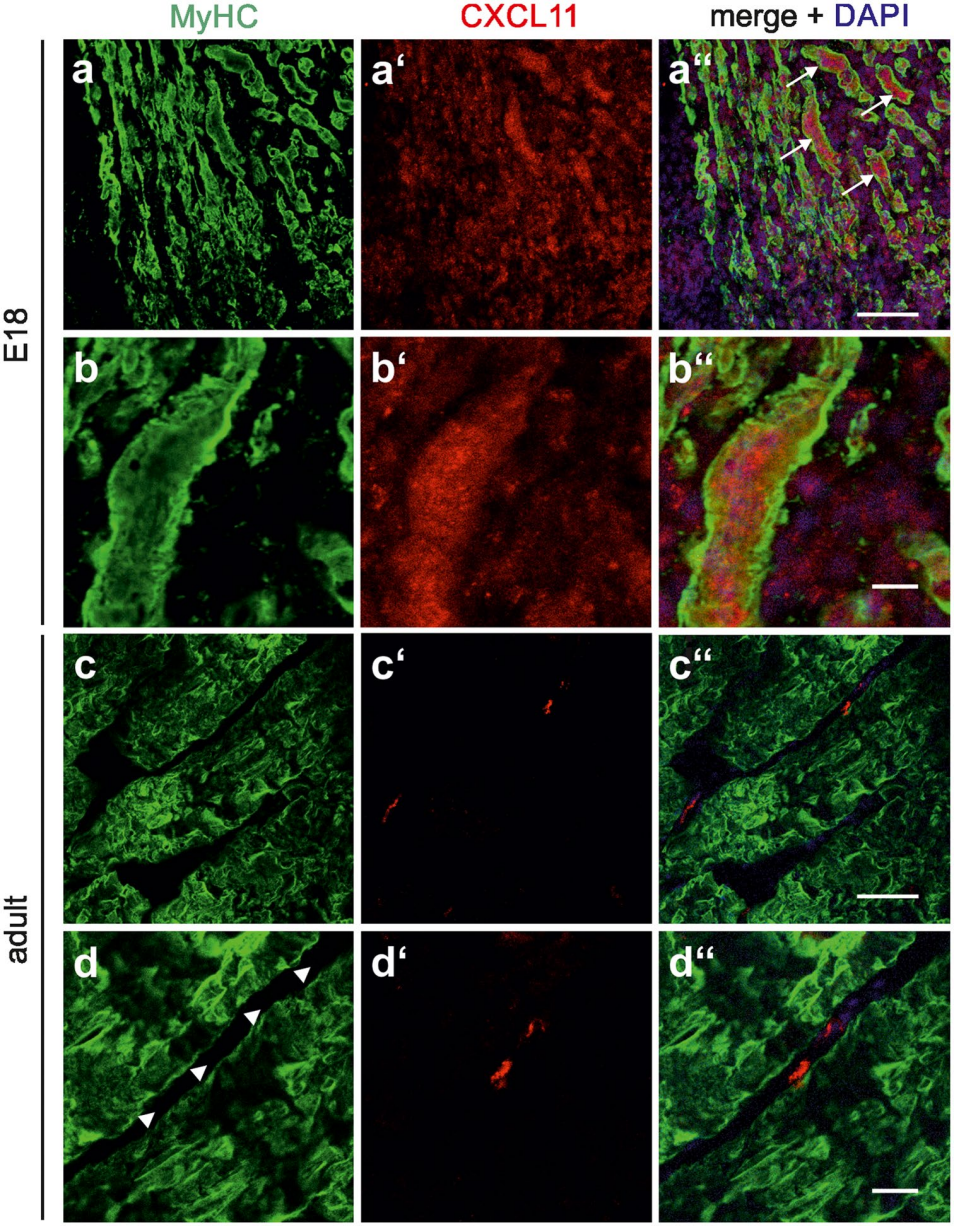
Localization of CXCL11 expression in developing and adult limb muscles. Sections were obtained from the quadriceps muscle of E18 **a**, **b** and adult rats **c**, **d** and double-labelled with antibodies against CXCL11 (red) and MyHC (green). Cell nuclei were visualized with DAPI. At each developmental stage, first row **a**, **c** and second row **b**, **d** show low and high magnification, respectively. **a″**–**d″** Merging of immunocytochemical stainings and DAPI. In E18 muscles, CXCL11-immunolabelling was associated with many muscle fibres (arrows in **a″**). Higher magnification **b″** confirmed the predominant intracellular localization of CXCL11 at this developmental stage. In adult muscles, CXCL11 expression was confined to cells within the endomysium/perimysium (arrow heads in **d**). Scale bars, **a** and **c**, 100 µm; **b** and **d**, 25 µm

Confirming and extending previous findings from RT-PCR analysis (Griffin et al. 2010), Western blotting allowed the detection of moderate levels of CXCL11 in C2C12 cells maintained under proliferation conditions (Fig. 1a; day 0). Following the switch to differentiation conditions, CXCL11 expression gradually increased by fourfold within 3 days (Fig. 1a'). It is of note that both ß-actin and GAPDH, routinely used to control for protein loading, were of limited use in these experiments. In fact, ß-actin levels were high in undifferentiated C2C12 cells and gradually declined with ongoing differentiation, whereas GAPDH expression increased with differentiation (Fig. 1a). The search for other non-regulated and, hence, more reliable loading controls remained unsuccessful; this issue is additionally exemplified for Erk in Supplementary Fig. 2. Consequently, care was taken to adjust all samples to identical protein levels prior to Western blotting. Analysis of the rat quadriceps muscle at different developmental stages further showed prominent CXCL11 levels at E18—the developmental stage at which secondary fibre formation takes place in rodents (Rubinstein and Kelly 1981)—with a sharp decline up to P3 and the virtual loss of CXCL11 into adulthood (Fig. 1b, b'). Double-labelling of sections of the E18 quadriceps muscle with CXCL11 and MyHC antibodies further revealed that CXCL11 expression was confined to MyHC-immunoreactive muscle fibres and prevailed in intracellular structures (Fig. 2a, b). By contrast, in the adult muscle, CXCL11 expression was virtually absent from muscle fibres but present in some endomysial/perimysial cells, presumably endothelial cells, immune cells and/or fibroblasts (Fig. 2c, d). Together these findings confirm that CXCL11 is expressed by myoblasts/myofibres mainly during active stages of skeletal muscle formation and, thus, seems to control muscle development in an autocrine/paracrine fashion.

### Expression of CXCL11 receptors in developing skeletal muscles

**Fig. 3:**
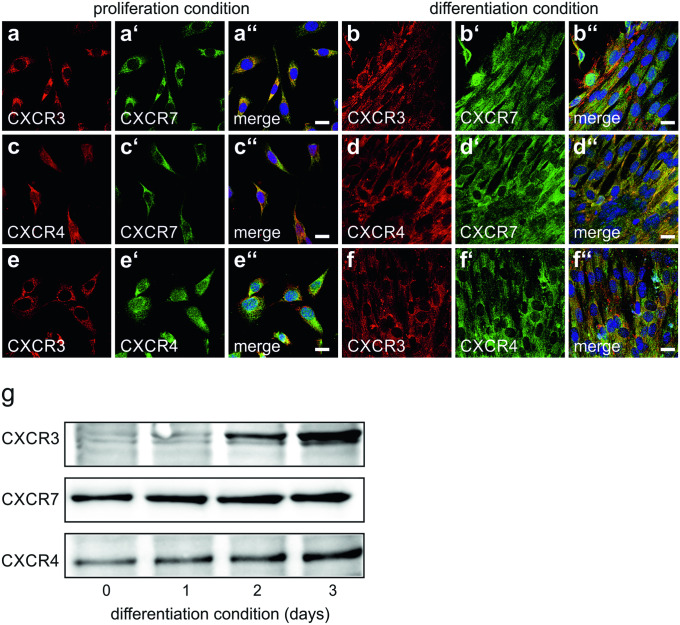
Expression and co-localization of chemokine receptors in C2C12 cells **a**–**f** C2C12 cells were maintained under either proliferation conditions **a**, **c**, **e** or differentiation conditions **b**, **d**, **f** for 3 days and double-labelled with antibodies against CXCR3 (bs-2209R), CXCR7, or CXCR4 as indicated. **a″**–**f″** Merging of immunocytochemical stainings and DAPI. Virtually all non-differentiated C2C12 cells expressed CXCR3 **a**, **e**. Expression of CXCR3 persisted in forming myotubes **b**, **f**. In addition to CXCR3, again virtually all non-differentiated C2C12 cells as well as forming mytotubes co-expressed CXCR7 **a″**, **b″** and CXCR4 **e″**, **f″**. Virtually all proliferating and differentiating cells further co-expressed CXCR4 and CXCR7 **c″**, **d″**. Scale bars, 10 µm. **g** Quantification of chemokine receptors expression in differentiating C2C12 cells. C2C12 cells were maintained for the indicated times with differentiation medium and analysed for CXCR3, CXCR7 and CXCR4 by Western blotting. Expression of the chemokine receptors increased with ongoing differentiation. Due to the lack of appropriate loading controls, care was taken to adjust samples to identical protein levels

We previously showed that differentiating myoblasts as well as muscle fibres from adult muscle express CXCR7 (Hunger et al. 2012). It is, however, presently unknown whether myoblasts (or muscle fibres) would additionally express the alternate CXCL11 receptor, CXCR3. Immunocytochemistry demonstrated that under proliferation conditions, virtually all C2C12 cells were immunoreactive for CXCR3 (Fig. 3a, e; antibody, bs-2209R). Double-labelling with different combinations of CXCR3, CXCR7 and CXCR4 antibodies additionally showed that again virtually all C2C12 cells co-expressed CXCR3, CXCR7, and CXCR4 (Fig. 3a'', c'', e''). Co-expression of CXCR3, CXCR7 and CXCR4 persisted in most C2C12 myotubes, which typically form when C2C12 cells are maintained under differentiation conditions (Fig. 3b'', d', f''). Quantification of chemokine receptor protein levels by Western blot analysis demonstrated that expression of CXCR3, CXCR7 and CXCR4 increased in differentiating C2C12 cells with a most pronounced increase of CXCR3 (Fig. 3g).

**Fig. 4:**
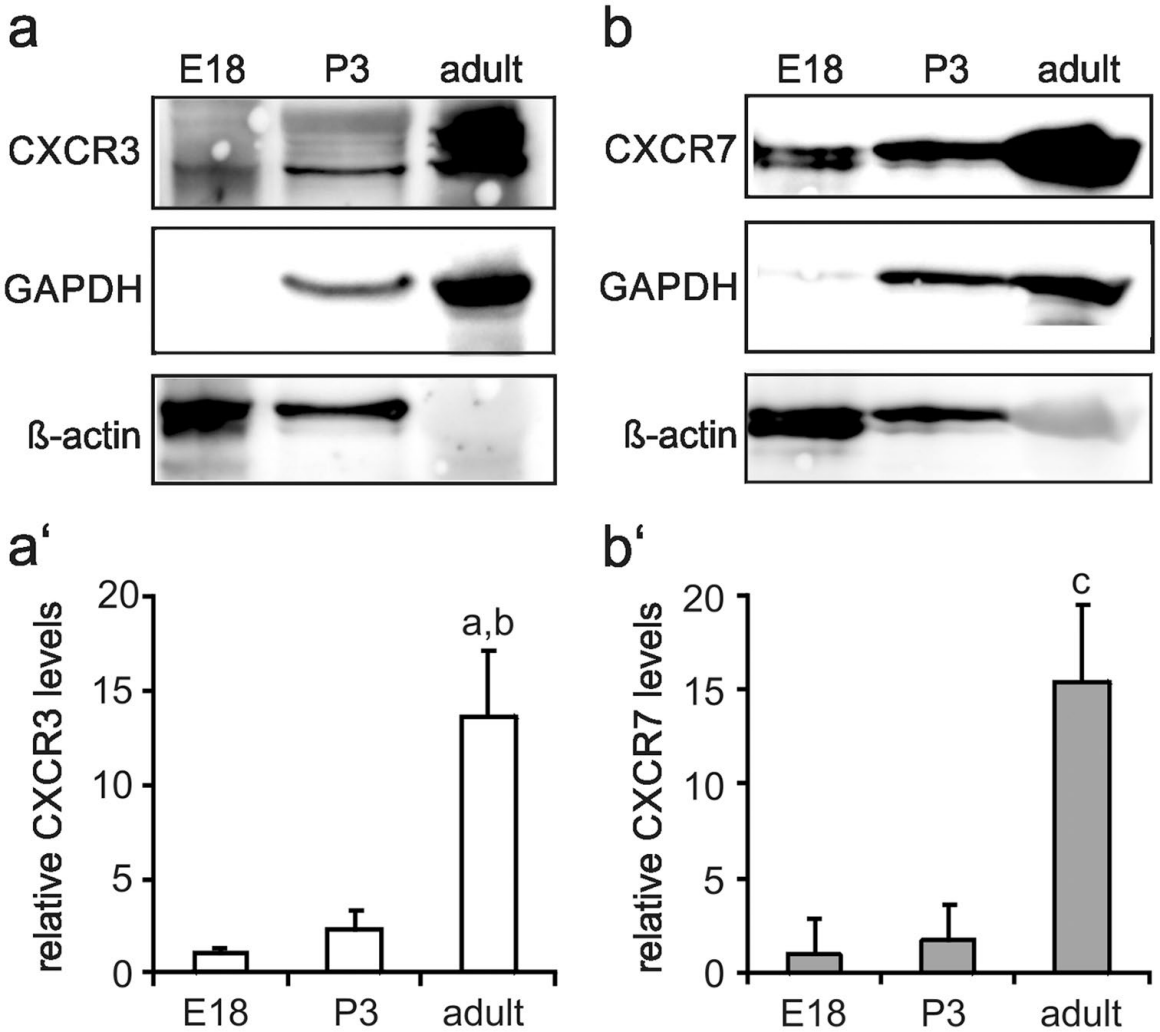
Expression levels of CXCL11 receptors in developing and adult limb muscles **a**, **b** Western blotting allowed the detection of the CXCL11 receptors, **a** CXCR3 and **b** CXCR7, in the quadriceps muscle of embryonic to adult rats with a most prominent increase in expression levels between P3 and adulthood. **a′**, **b′** Relative protein levels of **a′** CXCR3 and **b′** CXCR7 (mean ± SD, *n* = 3) as determined by densitometric analysis of immnoreactive protein bands. Protein levels present at E18 were set to 1. Note again that ß-actin and GAPDH, used as loading controls, are developmentally regulated (see Fig. 1). ^a^*p* < 0.001, adult vs E18; ^b^*p* < 0.01, adult vs. P3; ^c^*p* < 0.001, adult vs. E18 or P3

**Fig. 5:**
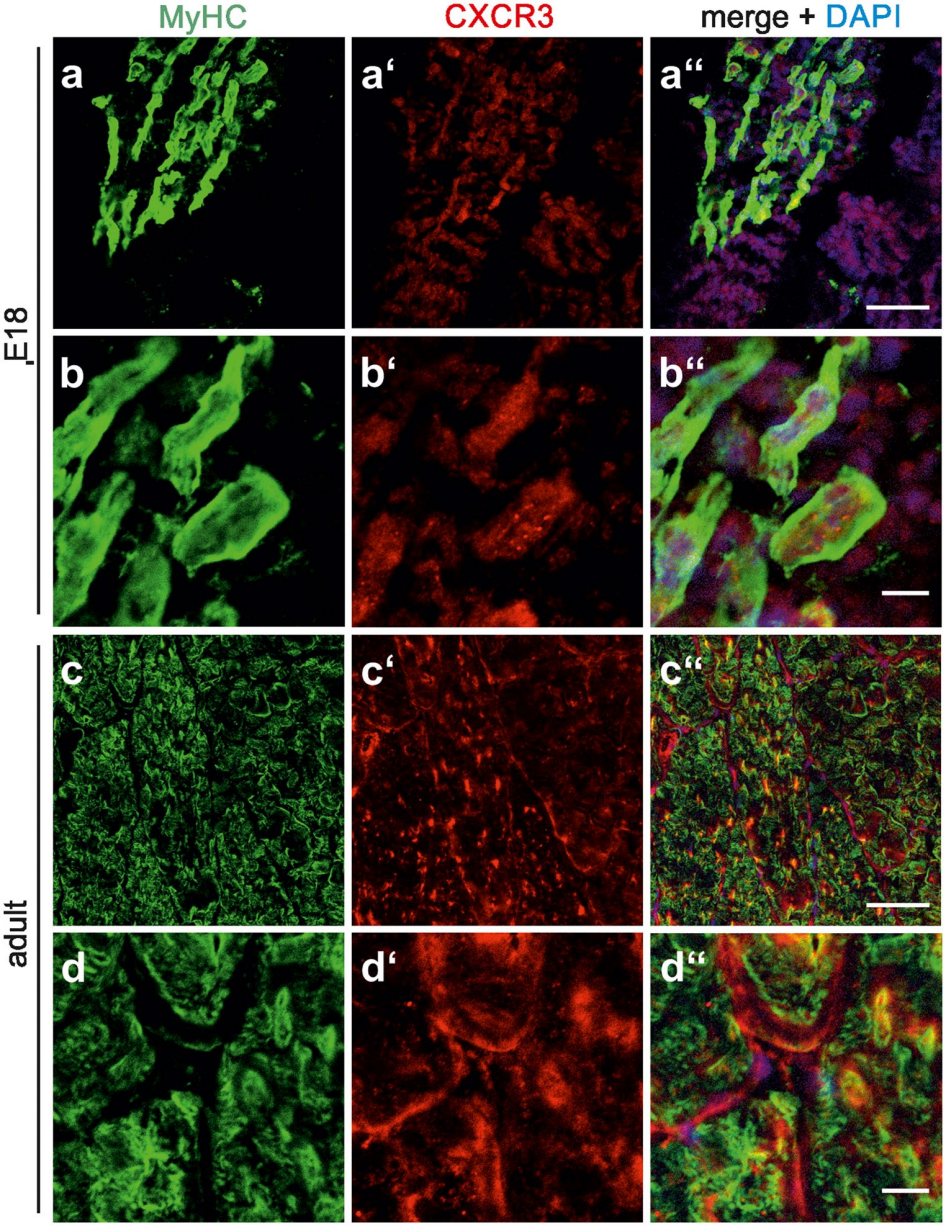
Localization of CXCR3 in developing and adult limb muscles. **a**–**d''** Sections of the quadriceps muscle from E18 **a**, a', **b, b'** and adult rats **c, c'**, **d, d'** were double-labelled with antibodies against CXCR3 (red; bs-2209R) and MyHC (green) as indicated. **a″**–**d″** Merging of immunocytochemical stainings and DAPI. At each developmental stage, first row **a**, **c** and second row **b**, **d** show low and high magnification, respectively. In the embryonic muscle, CXCR3 is primarily located within MyHC-positive muscle fibres **b″**, whereas in adult muscles CXCR3 is associated with both the sarcolemma and intracellular structures **d″**. Scale bars, **a** and **c**, 100 µm; **b** and **d**, 25 µm

Western blotting further confirmed the presence of moderate levels of CXCR3 (antibody, bs-2209R) in the quadriceps muscle of E18 and P3 rats with a prominent increase in CXCR3 expression into adulthood (Fig. 4a, a'). A similar expression pattern applied for CXCR7, which served as a positive control (Fig. 4b, b'; Hunger et al. 2012). Double-labelling of sections of the quadriceps muscle with CXCR3 (antibody, bs-2209R) and MyHC antibodies further unravelled that in both the E18 and adult muscle, CXCR3 expression is primarily associated with muscle fibres (Fig. 5). In addition, the experiments showed that at E18 CXCR3 was mainly confined to intracellular structures, whereas CXCR3 was predominantly, although not exclusively, present at the surface of adult muscle fibres (Fig. 5). This is reminiscent of the previously observed expression pattern and subcellular location of CXCR7 in developing limb muscles. Again, CXCR7 expression is mainly associated with muscle fibres and increases up to adulthood (Hunger et al. 2012; Puchert et al. 2016). Moreover, CXCR7 shows a primarily intracellular location in embryonic muscle fibres and prevails at the sarcolemma of adult muscles. In accordance with the observed expression of CXCR3 in skeletal muscle fibres, Feferman et al. (2005) previously reported the expression of CXCR3 on adult muscle fibres from healthy humans, while in other studies CXCR3 expression was described to prevail in invading (immune) cells or activated endothelia of inflamed muscles (De Paepe et al. 2005, 2012; Raju et al. 2003; Fall et al. 2005; Lv et al. 2018). With respect to these controversial observations, we wish to note that we analysed CXCR3 expression in adult rodent muscle fibres by using a CXCR3 antibody with confirmed specificity (Supplementary Fig. 1). These present observations together with our previous findings (Hunger et al. 2012) establish that in skeletal muscle fibres expression of the CXCL11 receptors, CXCR3 and CXCR7, extends from embryonic/prenatal development into adulthood.

### Evaluating the role of the CXCL11 system in muscle development

Steps of skeletal muscle development affected by chemokines are (1) myoblast/muscle stem cell proliferation and migration, (2) myogenic differentiation and (3) formation of muscle fibres/myotubes (Ödemis et al. 2007; Griffin et al. 2010; Melchionna et al. 2010; Vasyutina et al. 2005). To assess putative mitogenic effects, C2C12 cells, previously treated with CXCL11 (100 ng/ml) for 24 h, were assayed for BrdU incorporation (24 h). FACS analysis showed that 6.3 ± 0.4% and 4.5 ± 0.5% of the cells incorporated BrdU in the presence and absence of CXCL11, respectively. In the presence of high-serum (10% FCS) DMEM about 30% of the cells showed uptake of BrdU (Supplementary Fig. 5). In complementary experiments, C2C12 cells were treated with CXCL11 for 48 h and subsequently analysed for expression of the proliferation marker, Ki67, by RT-PCR. Relative Ki67 mRNA levels were 1.0 ± 0.06 and 1.29 ± 0.20 (*n* = 3–4) in the absence and presence of CXCL11, respectively. Collectively, these findings demonstrate that CXCL11 is without effects on the proliferation of C2C12 cells.

**Fig. 6:**
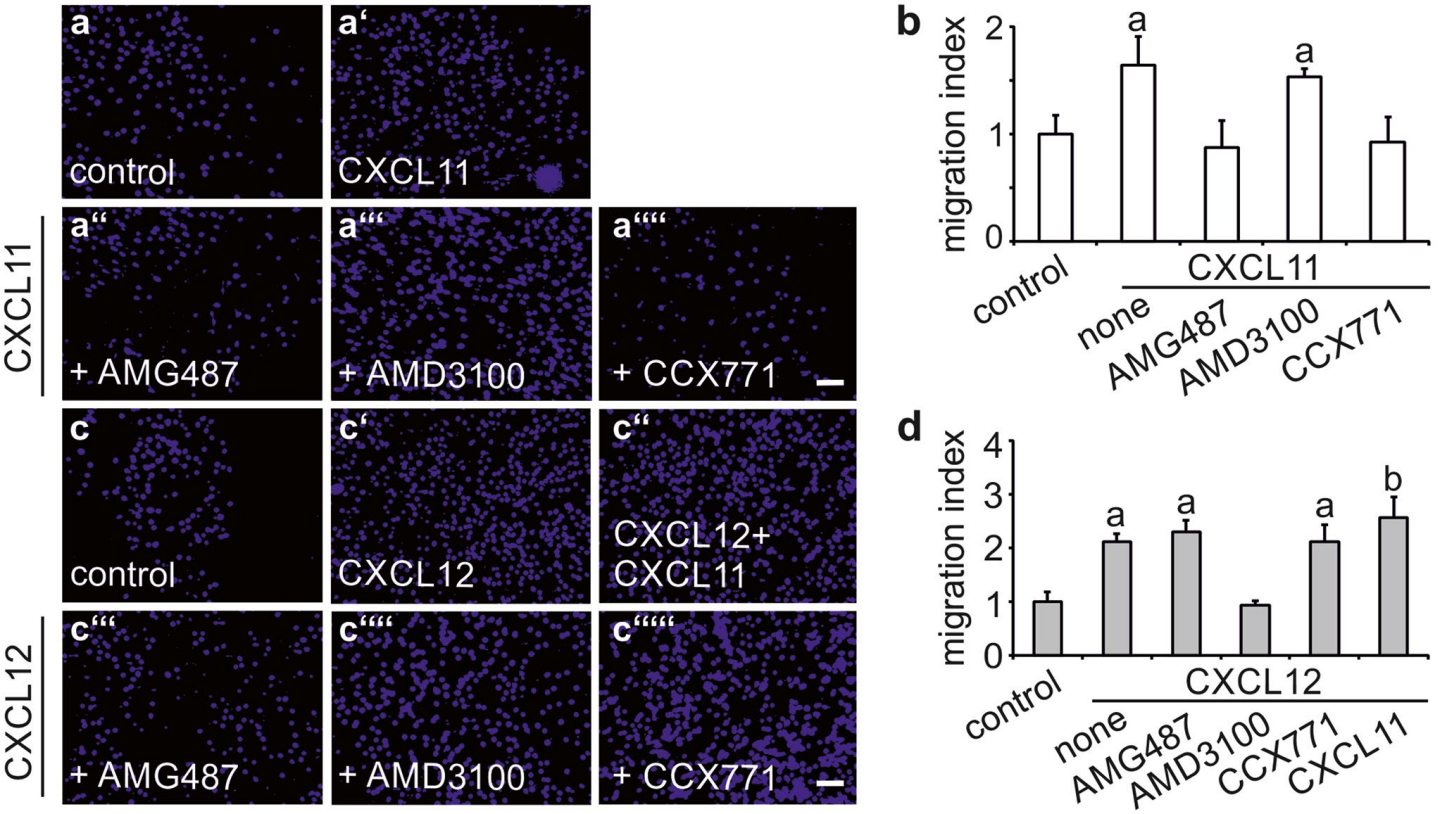
Comparison of the chemotactic responses of C2C12 cells to CXCL11 and CXCL12 **a**, **c** Chemotactic responses of C2C12 cells to **a** CXCL11 (100 ng/ml) and **c** CXCL12 (100 ng/ml) were assessed in a modified Boyden chamber as described under Materials and Methods. Cells were treated with the CXCR3 antagonist, **a″**, **c‴** AMG487 (10 µm), the CXCR4 antagonist, **a‴**, **c″″** AMD3100 (10 µM) or the CXCR7 antagonist, **a″″**, **c″″′** CCX771 (100 nM), 1 h prior to analysis. Photographs were taken at 50× from membranes following removal of non-migrated cells and staining of migrated cells with DAPI. **b, d** Migration index as defined by the ratio of cells migrated in the presence and absence of **b** CXCL11 and **d** CXCL12. Bars show mean ± SD (*n* = 5–8). While CXCL11-induced migration of C2C12 cells was sensitive to AMG487 and CCX771, CXCL12-induced migration was only sensitive to AMD3100. ^a^*p* < 0.001, treatment vs. untreated control; ^b^*p* < 0.01, double treatment vs. single treatment. Scale bars, 100 µm

Migratory responses of C2C12 cells to CXCL11 were assessed in a modified Boyden chamber (Ödemis et al. 2007). CXCL11 induced migration of undifferentiated C2C12 cells with a potency close to that of CXCL12, which served as a positive control (Fig. 6a', b, c', d; Ödemis et al. 2007). Moreover, the effects of CXCL11 and CXCL12 on the migration of C2C12 cells were partially additive (Fig. 6c'', d). Treating C2C12 cells 1 h prior to analysis with either the CXCR3 antagonist, AMG487, or the CXCR7 antagonist, CCX771, completely prevented CXCL11-induced cell migration (Fig. 6a, b). Chemotactic responses, however, were not affected by the CXCR4 antagonist, AMD3100 (Fig. 6a, b). Vice versa, CXCL12-induced migration of C2C12 cells was prevented by AMD3100 but remained unaffected by CCX771 and AMG487 (Fig. 6c, d). These findings establish that CXCL11 and CXCL12 control migration of C2C12 cells by signalling through CXCR3/CXCR7 and CXCR4, respectively.

**Fig. 7:**
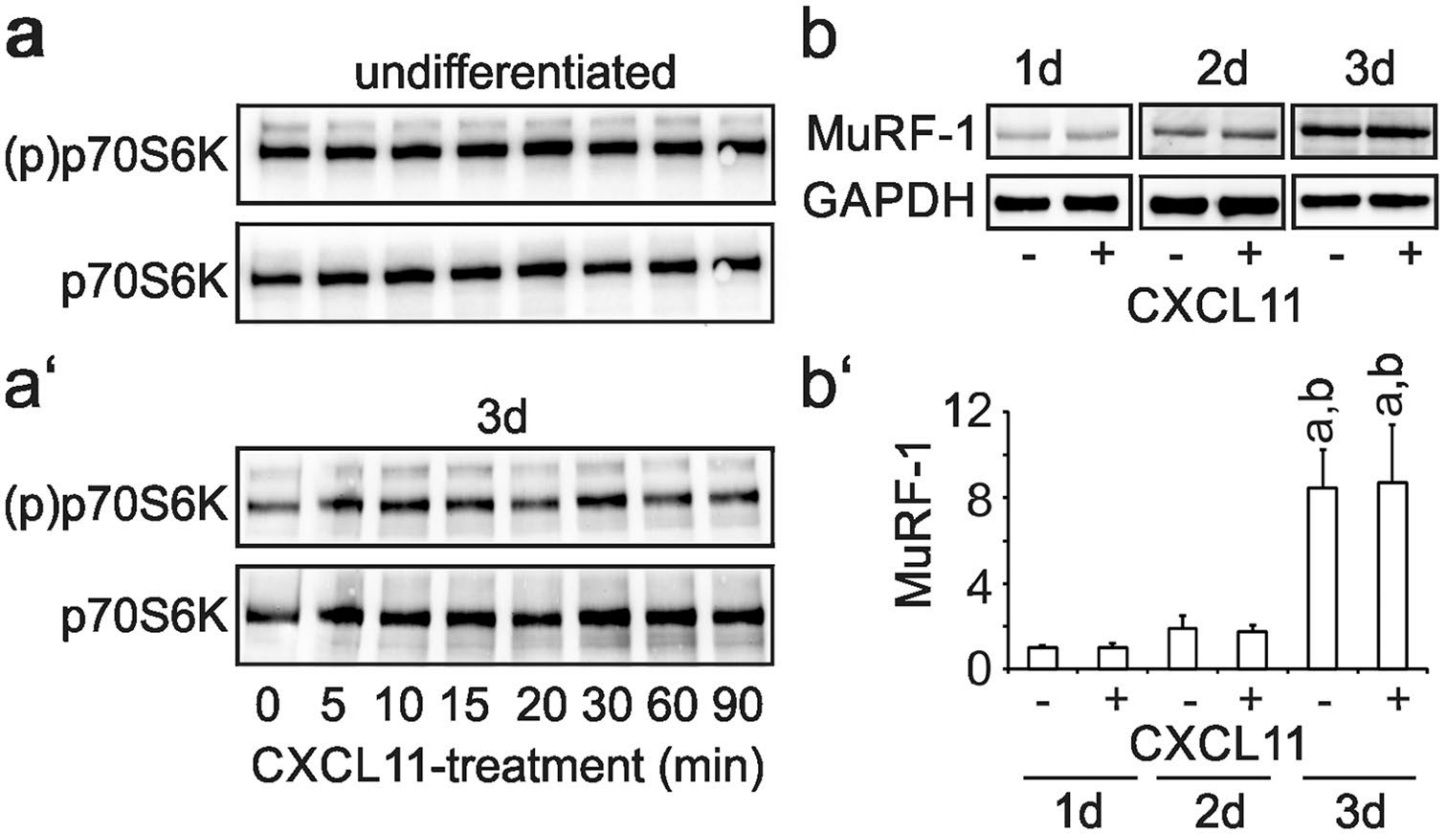
CXCL11 does not affect anabolic and catabolic markers in C2C12 cells. **a** Undifferentiated C2C12 cells or **a′** cells differentiated for 3 days were treated with CXCL11 (100 ng/ml) for the indicated time and subsequently analysed for phosphorylated (activated) p70S6K by Western blotting using phospho-specific antibodies. To control for protein loading, blots were additionally stained with antibodies recognizing p70S6K independent of its phosphorylation status. CXCL11 does neither activate p70S6K in undifferentiated nor differentiated C2C12 cells. **b** C2C12 cells differentiated for 3 days with low serum DMEM were maintained for another 1–3 days with low serum DMEM in the absence or presence of CXCL11 (100 ng/ml) without further medium change (starvation condition) but daily renewal of CXCL11 and analysed for MuRF-1 expression by Western blotting. GAPDH served as a loading control. **b′** Results from densitometric analysis of immunoreactive protein bands (mean ± SD, *n* = 3–4) corrected for protein loading. MuRF-1 expression continuously increased in starving C2C12 cells/myotubes and remained unaffected by CXCL11. ^a^*p* < 0.001, day 3 vs day 1; ^b^*p* < 0.001, day 3 vs. day 2

Putative effects of CXCL11 on myogenic differentiation were assessed by determining expression of the myogenic markers, myogenin and MyHC. Western blot analysis demonstrated a sharp increase in myogenin and MyHC levels in C2C12 cells maintained for 3 days under differentiation conditions (Supplementary Fig. 3). Expression levels of both myogenic markers remained unchanged in C2C12 cells maintained for 3 days with differentiation medium additionally supplemented with CXCL11 (100 ng/ml; Supplementary Fig. 3). CXCL11 further had no obvious effects on gross numbers and growth of myotubes, which form when C2C12 cells are maintained under differentiation conditions (Supplementary Fig. 4). The average diameter of mytotubes was 14.0 ± 8.8 µm and 14.4 ± 7.8 µm (average ± SD; *n* = 1000 per condition) when maintained for 3 days in the absence and presence of CXCL11 (100 ng/ml), respectively. Collectively, these observations favour the assumption that during development CXCL11 directs the formation of muscle anlagen by regulating myoblast migration.

### CXCL11 is not essential for the maintenance of adult muscles

We recently provided evidence that CXCL12 is essential for maintenance of adult muscles by affecting the anabolic mTOR-p70S6K pathway as well as MuRF-1-dependent catabolic mechanisms (Puchert et al. 2016). To assess putative effects of CXCL11 on the activity of p70S6K, C2C12 cells, maintained either under proliferation conditions or under differentiation conditions for 3 days, were treated with CXCL11 5 for 90 min (100 ng/ml) and subsequently analysed for activated (phosphorylated) p70S6K by Western blotting, using phospho-specific antibodies. CXCL11 failed to activate p70S6K at all time points examined (Fig. 7a, a'). To assess putative effects of CXCL11 on MuRF-1 expression, cultures of C2C12 cells were switched to differentiation medium for 3 days. Formed myotubes were subsequently maintained with differentiation medium containing CXCL11 (100 ng/ml) for another 1–3 days without any further medium change (starvation condition) but daily addition of fresh CXCL11. Subsequent Western blot analysis revealed that expression of MuRF-1 gradually increased up to day 3 of starvation and further showed that at all time points MuRF-1 expression levels remained unaffected by CXCL11 (Fig. 7b, b').

## Discussion

Previous work from our group and others discovered that chemokine CXCL12 affects multiple steps of limb myogenesis, including migration, proliferation and differentiation of myoblasts as well as formation of muscle fibres / myotubes (Hunger et al. 2012; Melchionna et al. 2010; Griffin et al. 2010; Bae et al. 2008; Ödemis et al. 2007). In addition, this work demonstrated that CXCL12 also controls maintenance and growth of adult skeletal muscles (Puchert et al. 2016; Martinelli et al. 2016). Although myogenic cells express CXCR7 and CXCR4, our previous work further established that CXCL12 signalling in muscle cells selectively occurs through CXCR4 (Puchert et al. 2016; Hunger et al.  2012). Since CXCR7 also binds CXCL11, which in turn uses CXCR3 as its second chemokine receptor (Singh et al. 2013), we have now asked whether the CXCL11/CXCR7/CXCR3 axis would likewise affect skeletal muscle development and maintenance. We observed that comparable with CXCR7, CXCR3 expression occurs in myoblasts and continues into mature muscle fibres, whereas expression of CXCL11 is restricted to skeletal muscle development. In addition, we show that CXCL11 controls migration of myoblasts by signalling through CXCR3 and CXCR7.

Several of our present findings underline a prominent role of CXCL11 in recruiting myoblasts/myogenic stem cells to develop muscle anlagen in order to fuse with growing muscle fibres. Firstly, we identified CXCL11 as a potent chemoattractant for C2C12 cells, a well-established model for myoblasts/myogenic stem cells. Secondly, we demonstrated that CXCL11 is expressed in late embryonic rat limb muscles, the stage when secondary fibre formation takes place (Rubinstein and Kelly 1981). Whether this CXCL11-dependent mechanism is reactivated during regeneration of injured skeletal muscles awaits experimental clarification. The previous demonstration that muscle satellite cells express CXCR3 (Raju et al. 2003) together with the finding that CXCL11 is re-expressed in necrotic muscle fibres (de Paepe et al. 2012) currently favours such a role.

The use of selective receptor antagonists demonstrated that C2C12 cells require both, CXCR3 and CXCR7, to respond to CXCL11 with cell migration. There is precedence for the use of a combination of chemokine receptors to control distinct cell functions (see for example Lipfert et al. 2013; Chen et al. 2015; Puchert et al. 2018). Based on the available data, we are presently unable to discern whether the observed co-operation between CXCR3 and CXCR7 depends on receptor heterodimerization (Martínez-Muñoz et al. 2018) or the convergence of down-stream receptor signalling. Extending our previous findings of a prime role of CXCR4 in the effects of CXCL12 on differentiation of C2C12 cells (Hunger et al. 2012), our experiments now further revealed that CXCL12 induces migration of C2C12 cells by acting solely through CXCR4. Interestingly, CXCL11 in combination with CXCL12 additively promoted myoblast migration. We currently consider this observation as an indication that CXCR3/CXCR7 and CXCR4 control migration of C2C12 cells by different down-stream signalling pathways. The partial redundancy of the effects of CXC11/CXCR3/CXCR7 and CXCL12/CXCR4 on myoblast migration could also explain why the absence of CXCL11 from C57BL/6 mice (natural null mutants) (Sierro et al. 2007) is not associated with obvious defects in skeletal muscle development. It is however equally possible that in these animals other CXCR3 ligands compensate for CXCL11 during myogenesis as shown for other types of cells/cellular systems, e.g., T cells (Groom and Luster 2011).

Controversy surrounds the issue whether CXCR3 is expressed by adult muscle fibres. Whereas Feferman et al. (2005) demonstrated expression of CXCR3 in skeletal muscle fibres from healthy humans, several other studies just reported CXCR3 expression in invading immune cells or activated endothelium of diseased muscles (De Paepe et al. 2005, 2012; Raju et al. 2003; Fall et al. 2005; Lv et al. 2018; Kim et al. 2014). It is of note that these studies primarily focused on muscle inflammation and since activated immune and endothelial cells express very high amounts of CXCR3 (Singh et al. 2013; Müller et al. 2010), the weaker expression of CXCR3 in (healthy) muscle tissue might have been neglected or just overseen. In our study, immunohistochemistry using a CXCR3 antibody with proven specificity demonstrated the persistent expression of CXCR3 in fibres of the adult rat quadriceps muscle as well as in forming C2C12 myotubes. These findings, however, do not dismiss the possibility that CXCR3 expression in mature muscle fibres only occur in distinct species. In a previous work we already demonstrated that developing and adult muscle fibres additionally express CXCR7 (Hunger et al. 2012). Despite the continuous expression of CXCR3 and CXCR7 in skeletal muscle fibres, CXCL11 is widely absent in adult muscle tissue. Moreover, treatment of C2C12 cells with CXCL11 remained without effects on cell proliferation; the expression of the myogenic markers, myogenin and MyHC; and the anabolic and catabolic markers, p70S6K, and MuRF-1, respectively. This obvious failure of CXCL11 to affect differentiation and maintenance of myoblasts/muscle fibres, however, disregards the possibility that muscular CXCR3 or CXCR7 might be activated by additional chemokines/ligands binding only one of these receptors. Candidates include the CXCR3 ligands, CXCL9 and CXCL10; the latter one was recently found to induce differentiation of cultured human myoblasts (Deyhle et al. 2018). Additional ligands for CXCR7 are macrophage inhibitory factor (MIF), adrenomedullin (ADM) and bovine adrenal medulla 22 (BAM22) (Wang et al. 2018). While MIF reportedly regulates proliferation and differentiation of myoblasts (Wen et al. 2016), effects of the other ligands on skeletal muscles are presently unknown. It is worth mentioning that several of the aforementioned CXCR3 and CXCR7 ligands, such as CXCL9, CXCL10 and MIF, are increased in skeletal muscles following injury (De Paepe et al. 2008; Kim et al. 2014; Limongi 2015; Reimann et al. 2010) and, via interacting with their respective receptors persistently present on muscle fibres, might regulate muscle immune responses and/or muscle regeneration.

Collectively, our findings identify the CXCL11-CXCR3-CXCR7 axis as part of the chemokine network controlling skeletal muscle development. In addition, the findings favour the existence of a previously unrecognized crosstalk between the CXCL11- and CXCL12 systems, which could essentially interfere with development, maintenance and/or regeneration of skeletal muscles.

## Supplementary Information

Below is the link to the electronic supplementary material.

Supplementary file1 (PDF 423 KB)
