# Supplementary material for: Identification of CXCL11 as part of chemokine network controlling skeletal muscle development
Source: Cell Tissue Res. 2021 Jan 27;384(2):499–511. doi: 10.1007/s00441-020-03398-0 (PMC8141492; doi:10.1007/s00441-020-03398-0)
Supplement: Supplementary file 1 — Supplementary file1 (PDF 423 KB) [file 441_2020_3398_MOESM1_ESM.pdf]

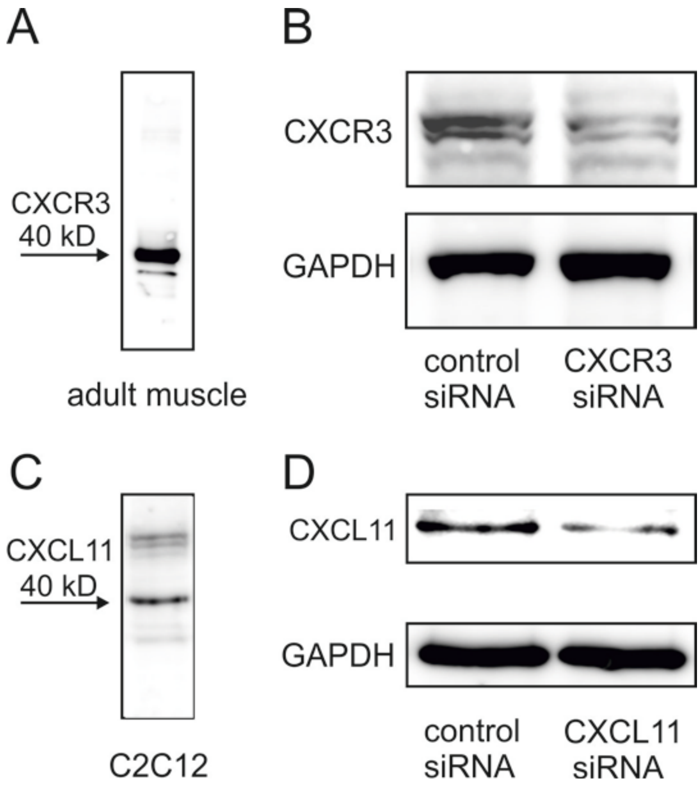

Supplementary Figure 1

Characterization of the specificity of CXCR3 and CXCL11 antibodies.

(A) Western blot from adult muscle tissue showing a major CXCR3-immunoreactive protein band with the approximate molecular weight of 40 kD. (B) Transfection of C2C12 cells with CXCR3 siRNA reduces the CXCR3-immunoreactive protein band in average by 73.7+6.6% (n = 3) after 2d when compared to cells transfected with control siRNA, hence, confirming the specificity of the antibody. (C) Western blot analysis of C2C12 cells, maintained for 3d under differentiation conditions, identifies an immunoreactive protein band with a molecular weight of 40 kD, which as shown in (D) is decreased (75.8+12.4%, n=3) 3 d following transfection of the cells with CXCL11 siRNA.

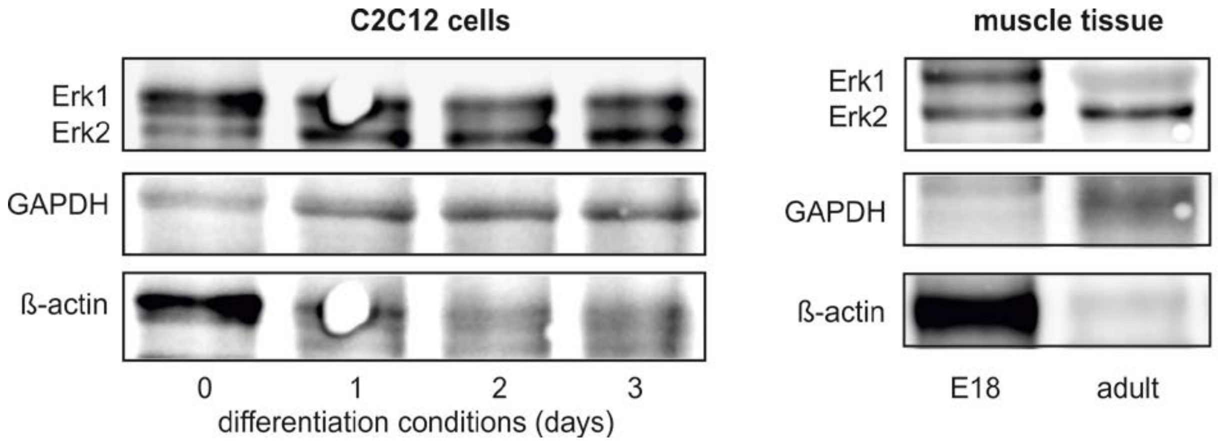

Supplementary Figure 2
Characterization of the suitability of Erk as a loading control.

Lysates were prepared from C2C12 cells maintained for the indicated times under differentiation conditions (DMEM + 1% FCS) as well as from E18 and adult rat quadriceps muscles. Following adjustment to identical protein levels, lysates were analyzed for expression levels of Erk (Erk1, Erk2), GAPDH, and ß-actin by Western blotting. Note that in addition GAPDH and ß-actin, expression of either Erk1 and/or Erk2 is developmentally regulated in C2C12 cells and skeletal muscles, hence, dismissing their use as a loading control for Western blot analysis.

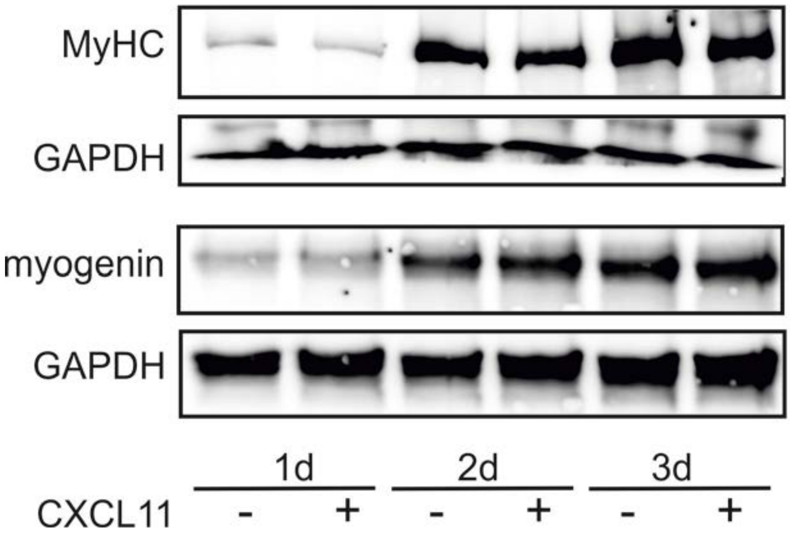

Supplementary Figure 3

CXCL11 does not interfere with myogenic differentiation.

Subconfluent cultures of C2C12 cells were switched to DMEM + 1% FCS (differentiation medium) additionally supplemented with CXCL11 (100 ng/ml) and analyzed for myosin heavy chain (MyHC) and myogenin expression by Western blotting after the indicated times. GAPDH served as loading control. Expression of MyHC and myogenin gradually increased with time. Expression of both myogenic markers remained unaffected by CXCL11. Experiments were replicated five times with similar findings.

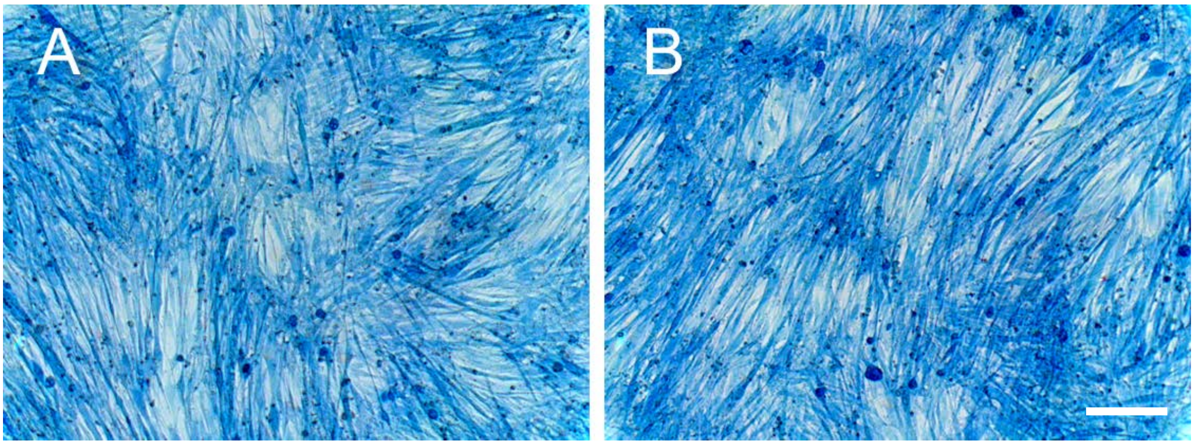

Supplementary Figure 4

CXCL11 does not affect myotube formation of C2C12 cells.

C2C12 cells were maintained for 3 days with differentiation medium (DMEM + 1% FCS) in (A) the absence or (B) presence of CXCL11 (100 ng/ml) and subsequently stained with methyl blue. Note that CXCL11 has no obvious effects on numbers of formed myotubes as well as on their diameters (see text). Scale bar, 200 µm.

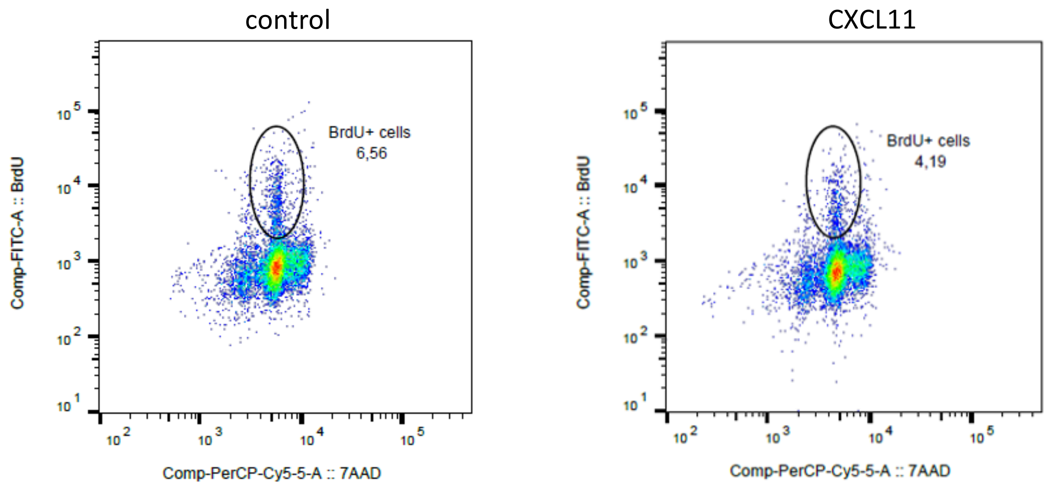

Supplementary Figure 5

CXCL11 has no obvious effects on the proliferation of C2C12 cells.

Cultured C2C12 cells were switched to serum-free DMEM supplemented with CXCL11 (100 ng/ml). After 24 h, cells were incubated for another 24 h with BrdU (10 mM). Following labeling with BrdU antibodies as well as with 7-AAD, cells were analyzed for BrdU-incorporation by FACS. CXCL11 had no statistically significant effects on BrdU-incorporation in C2C12 cells.
